# Phenotype prediction of Mohr-Tranebjaerg syndrome (MTS) by genetic analysis and initial auditory neuropathy

**DOI:** 10.1186/s12881-018-0741-3

**Published:** 2019-01-11

**Authors:** Hongyang Wang, Li Wang, Ju Yang, Linwei Yin, Lan Lan, Jin Li, Qiujing Zhang, Dayong Wang, Jing Guan, Qiuju Wang

**Affiliations:** 1 Institute of Otolaryngology, Chinese PLA General Hospital, Medical School of Chinese PLA, Beijing, 100853 China; 2 BGI-Shenzhen, Shenzhen, 518120 China

**Keywords:** Auditory neuropathy, Mohr-Tranebjaerg syndrome (MTS), *TIMM8A*

## Abstract

**Background:**

Mohr-Tranebjaerg syndrome (MTS) is a rare X-linked recessive neurodegenerative disorder resulting in early-onset hearing impairment, gradual dystonia and optic atrophy. MTS is caused by variations in the nuclear TIMM8A gene, which is involved in mitochondrial transport of metabolites. This study aimed to identify the pathogenic gene variations in three Chinese families associated with predicted MTS with or without X-linked agammaglobulinaemia.

**Methods:**

Otologic examinations, vestibular, neurological, optical and other clinical evaluations were conducted on the family members. Targeted genes capture combining next generation sequencing (NGS) was performed, and then Sanger sequencing was used to confirm the causative variation.

**Results:**

A novel variation, c.232_233insCAAT, in TIMM8A was identified as the pathogenic variation in one Chinese family. This variation co-segregated with the most frequent phenotypic deafness and was absent in the 1000 Genomes Project, ExAC and 1751 ethnicity-matched controls. Clinically, otological examinations illustrated the typical postsynaptic auditory neuropathy for the proband without the symptoms of dystonia or optic atrophy. MRI demonstrated abnormal small cochlear symmetric nerves, while the vestibular function appeared to be less influenced. Furthermore, we found another two TIMM8A variations, the deletion c.133_135delGAG and a copy number variation (CNV) including the TIMM8A gene, in two independent case, when we performed NGS on an auditory neuropathy population.

**Conclusion:**

We identified two novel variations in the TIMM8A gene (c.232_233insCAAT and c.133_135delGAG) and a CNV including the TIMM8A gene in three independent Chinese families with predicted MTS. To our knowledge, this is the first report of TIMM8A variations being identified in a Chinese population. Our results enrich the variation spectrum of TIMM8A and clinical heterogeneity of MTS. Genetic detection and diagnosis is a powerful tool for better understanding and managing syndromic hearing impairments, such as MTS, before they become full-blown.

**Electronic supplementary material:**

The online version of this article (10.1186/s12881-018-0741-3) contains supplementary material, which is available to authorized users.

## Background

Mohr-Tranebjaerg syndrome, also known as deafness-dystonia-optic neuronopathy (MTS/DDON, MIM304700) syndrome, is a rare X-linked recessive neurodegenerative disease. It is characterized by early-onset progressive auditory neuropathy followed by dystonia and optic atrophy in adolescence or adulthood. Additional psychiatric disorders have been reported frequently, such as dementia, irritability and mental retardation. First described in 1960 [1], MTS was mapped to Xq22 by Tranebjaerg in 1995 [2, 3], and the disease-causing variations were found in a gene named translocase of mitochondrial inner membrane 8A/deafness-dystonia peptide-1 (*TIMM8A*/*DDP1*) [4]. The *TIMM8A* gene encodes a 97-amino-acid protein, a translocase involved in the import of metabolite transporters from the cytoplasm into the mitochondrial inner membrane [5, 6].

MTS is a progressive disease associated with multiple systems throughout the patient’s life. However, it is not easy to discriminate it due to considerable variations both in the onset age and phenotypic expression [7]. To date, only 69 cases have been reported since the first MTS family was described in 1960. Most patients have suffered from deafness and subsequent dystonia for decades before they were diagnosed with MTS. Fortunately, molecular diagnosis through next-generation sequencing (NGS) has made it easier to diagnose deafness syndromes such as MTS, making diagnosis possible decades earlier, before all the phenotypes show. Targeted deafness genes combined with high-throughput sequencing can be regarded as a revolutionary technology with high speed and low cost to identify the pathogenic causes in a large number of syndromic hearing loss families [8, 9].

In this study, we performed variation screening of 127 known deafness-related genes in a young patient from Family 1 without any findings of common deafness-related gene screening. We identified a novel frameshift variation in the *TIMM8A* gene in this Chinese family with auditory neuropathy, who visited the outpatient department for deafness genetic consultation, as typical auditory neuropathy was the only phenotype in the proband at this stage. Furthermore, we performed targeted capture of 127 genes and NGS on another 167 patients diagnosed with auditory neuropathy and found another two *TIMM8A* variations, as well as agammaglobulinaemia in one positive case. To our knowledge, this is the first report of MTS cases recognized efficiently with NGS before clinical diagnosis.

## Methods

### Clinical evaluations

**Fig. 1:**
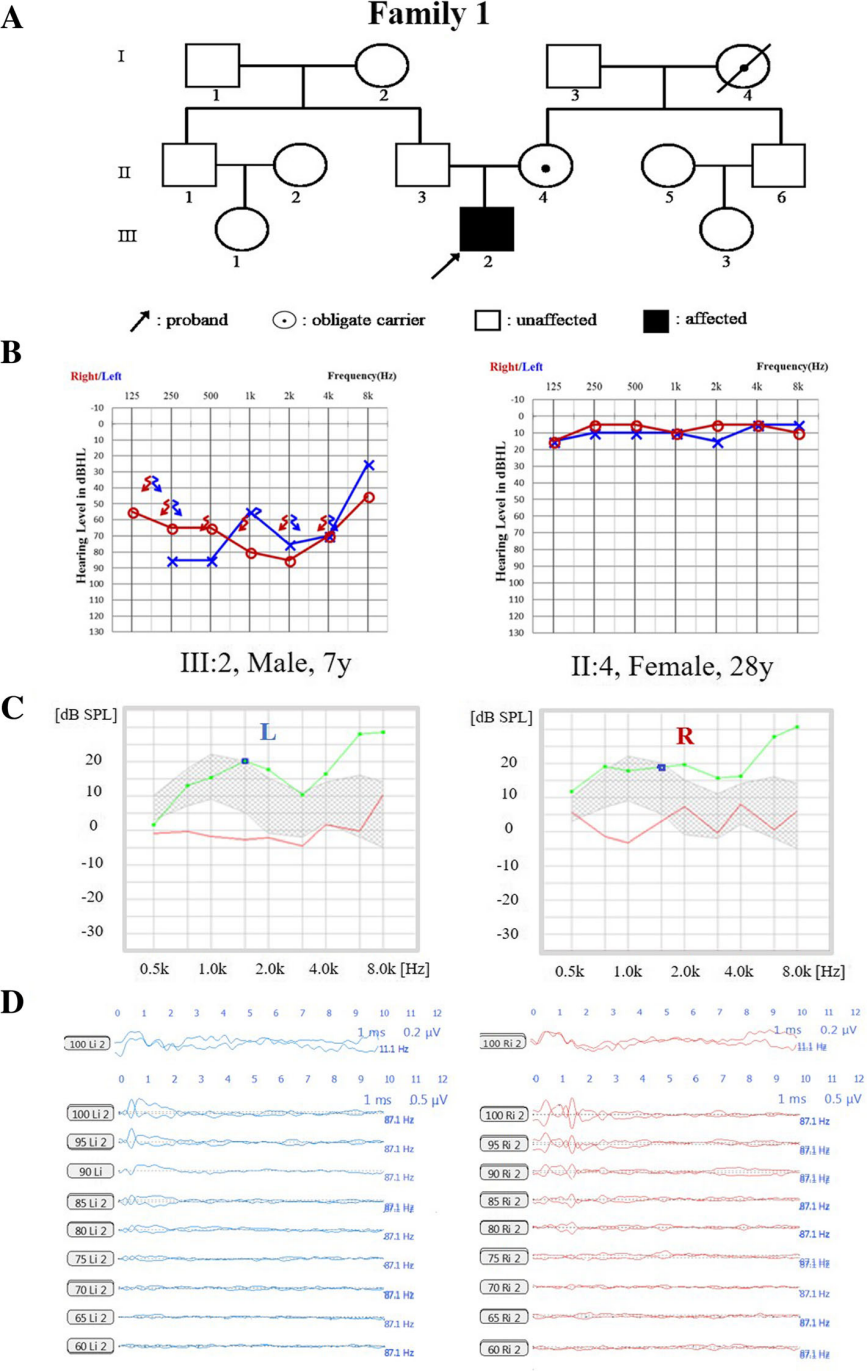
Pedigree and audiological phenotype of Family 1. **a** Pedigree of Family 1. The affected subject is coloured black, the proband is indicated by an arrow, and unaffected members with black spots indicate obligate carriers of the causative variation. **b** Audiograms of the proband (III:2) and his mother (II:4). **c** Normal DPOAE result of the proband. **d** Absence of ABR waves and present CM waves in the proband. L, Left, blue; R, Right, red

**Fig. 2:**
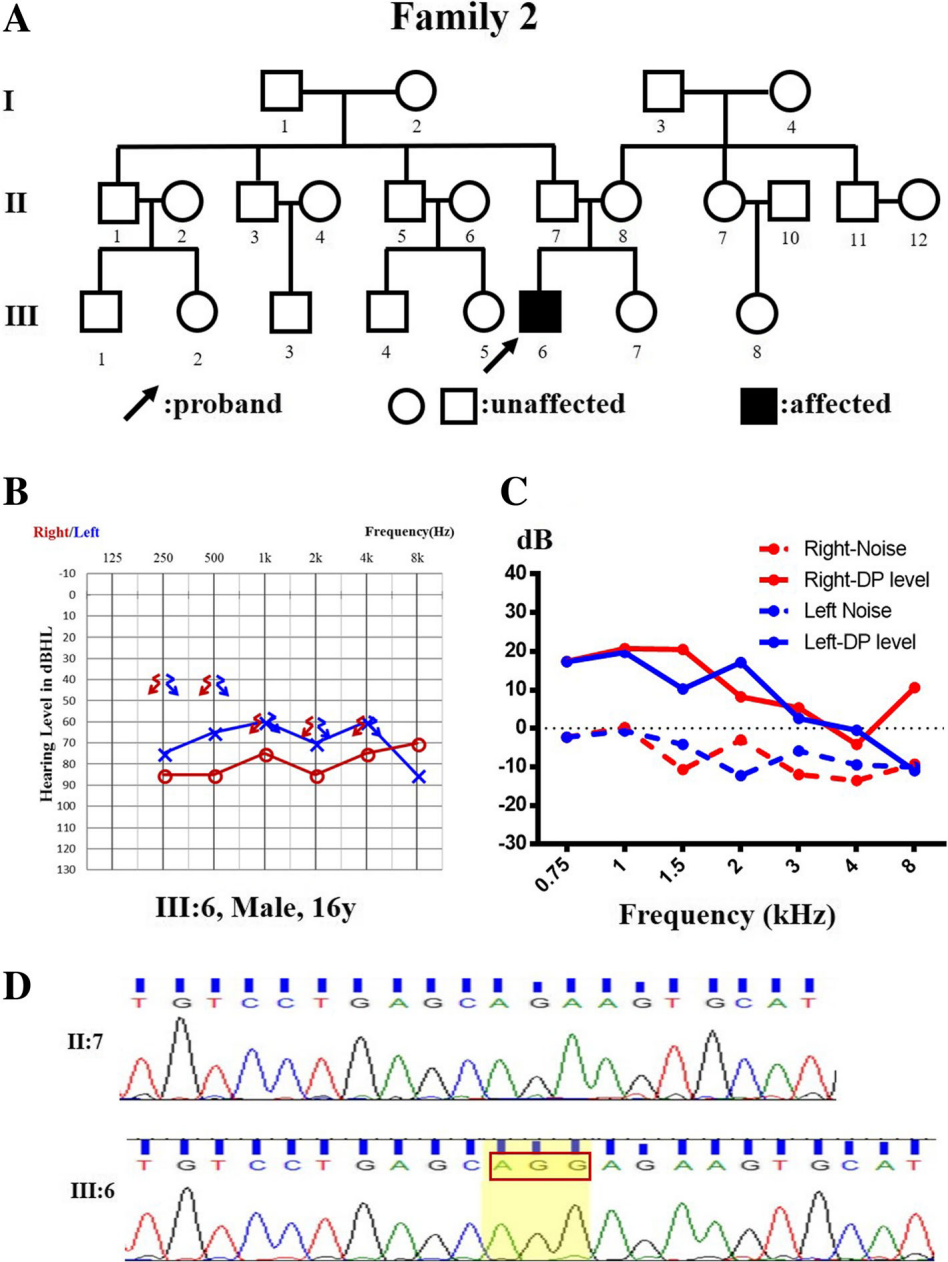
Pedigree, audiological phenotype and gene variation of Family 2. **a** Pedigree of Family 2. The affected subject is coloured black, and the proband is indicated by an arrow. **b** Audiograms of the proband (III:6). **c** Normal DPOAE result of the proband. **d** Sequencing chromatograms of TIMM8A showing the deletion in affected individuals (lower panel) compared with normal controls (upper panel). L, left, blue; R, right, red

**Fig. 3:**
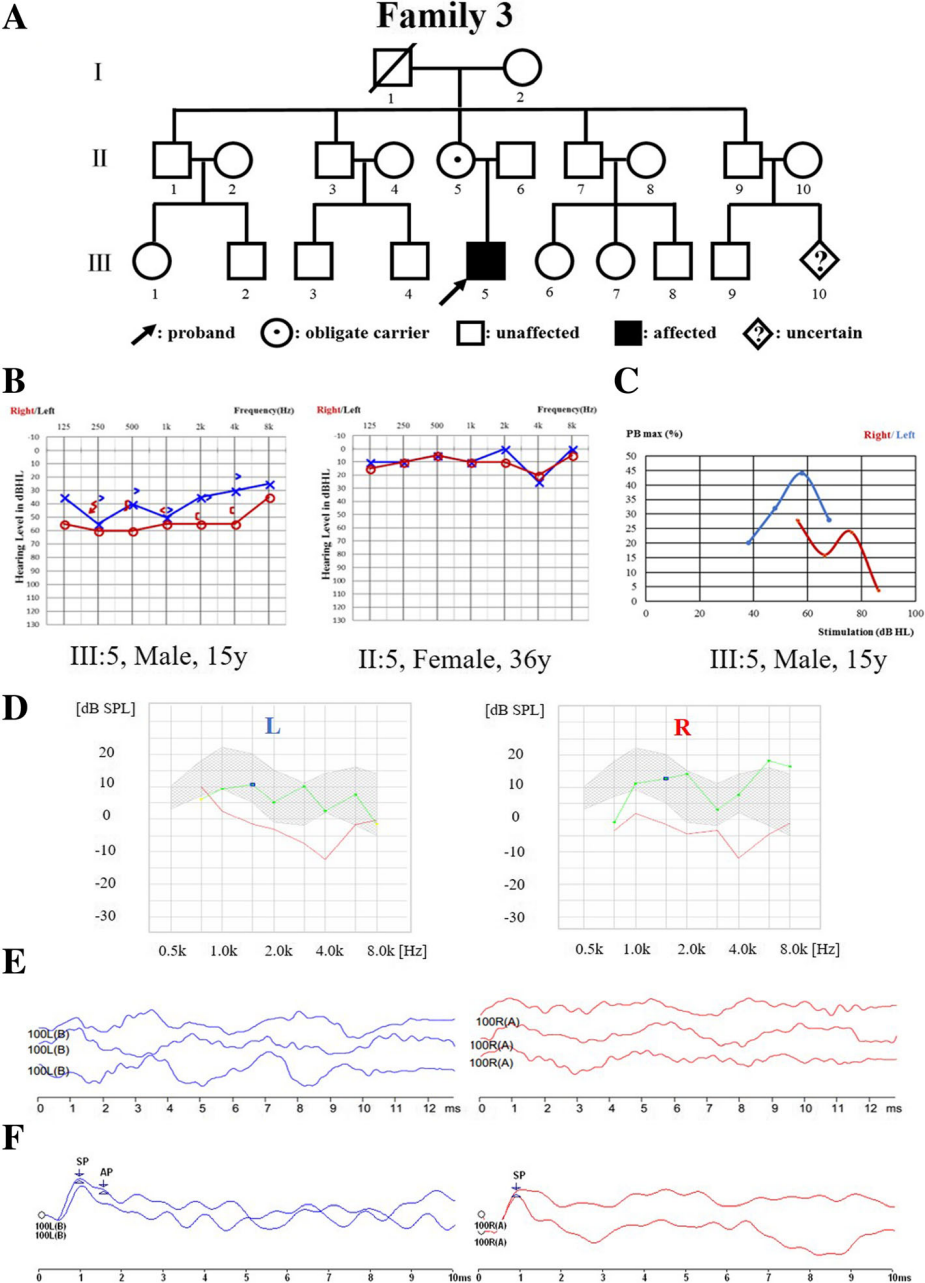
Pedigree and audiological phenotype of Family 3. **a** Pedigree of Family 3. The affected subject is coloured black, the proband is indicated by an arrow, unaffected members with black spots indicate obligate carriers of the causative variation, and a rhombus with a question mark is a subject with unknown gender. **b** Audiograms of the proband (III:5) and his mother (II:5). **c** Speech recognition score (SRS) of the proband upon different stimulations, showing different tendency from normal controls. **d** Normal DPOAE result of the proband. **e** Absence of ABR waves and present CM waves in the proband. **f** Electrocochleography (ECochG) showed abnormal -SP/AP > 0.4. L, left, blue; R, right, red

Three probands, from Family 1, Family 2 and Family 3, who were diagnosed with auditory neuropathy and their parents were recruited (Fig. 1a, Fig. 2a and Fig. 3a), written informed consents were obtained from the next of kin on the behalf of the minors/children participants involved in this study. This study was approved by the Ethics Committee of Chinese PLA General Hospital.

Their medical histories were collected by a questionnaire. Otological examinations, including otoscopy, immittance testing, pure-tone audiometric examination (PTA, Madsen Astera^2^, DENMARK), speech recognition score (SRS, Madsen Astera^2^, DENMARK), distortion product otoacoustic emission (DPOAE, Madsen Capella, DENMARK), click-evoked auditory brainstem response (ABR), cochlear microphonics (CM) and electrocochleography (ECochG) tests (SmartEp, USA or Neuro-Audio, Russia), were performed to evaluate the auditory conditions. The diagnosis of auditory neuropathy was made according to the results of DPOAE/CM and ABR tests. The hearing level was assessed at 125, 250, 500, 1000, 2000, 4000 and 8000 Hz by PTA. The brain magnetic resonance imaging (MRI) examination for the temporal bone of both ears was also performed on the proband. Vestibular function evaluation included vestibular-evoked myogenic potentials (ocular VEMP, oVEMP and cervical VEMP, cVEMP), oculomotor function tests, positional nystagmus tests, positioning nystagmus tests and bithermal caloric tests. The ocular examinations included visual acuity, perimetry, flash and pattern visual-evoked potential testing, and detailed stereoscopic fundoscopy. The neurological examination included regular physical examinations and electromyography.

### Targeted gene capture and NGS

Peripheral blood samples of these families were collected with consent. Genomic DNA was extracted from the whole blood of the probands and their parents (TIANGEN BIOTECH, BEIJING, CHINA). After the examination of DNA quality, Beijing Genomics Institute built the DNA libraries by following Illumina’s protocol, and then 127 known deafness-related genes, including exons, splicing sites and their flanking introns, were captured by using a custom probe and sequenced by the Illumina HiSeq2000 [10]. The paired-end reads generated by sequencing were aligned to the NCBI37/hg19 assembly by the BWA software, and variant calling was performed by GATK. The bioinformatics analysis method had been described in detail previously. To obtain rare variations, common variants and low-frequency variants in the 1000 Human Genomes Project database, HapMap Project database, ExAC database, EVS database and BGI in-house databases were excluded (0.5%). All remaining variants were considered to be rare variations and were annotated using Ensembl VEP, OMIM, MGI, Gene Ontology, HGNC gene annotation database and so on. Candidate variants were validated by Sanger sequencing.

### SNP array for CNV analysis

Genome-wide chromosome copy number anomalies were detected using Illumina’s InfiniumOmniZhongHua-8 DNA Analysis Bead Chip (200925710145_R03C01), with a resolution of 20 kb.

### Variant filtration, confirmation and modelling

Variants with allele frequencies higher than 5% in the 1000 Genomes Project and the local database were excluded. Splicing-site, frameshift and nonsense variants were taken into further consideration. Moreover, SIFT and PolyPhen2 software were used to evaluate the pathogenic possibility. Sanger sequencing was performed to establish the co-segregation of the candidate gene variations with the phenotype in the family members. A three-dimensional structure of TIMM8A was built by SWISS-MODEL (https://swissmodel.expasy.org/) and then visualized by Swiss-PdbViewer 4.1 (version 4.1, http://spdbv.vital-it.ch/).

### Overview of MTS cases reported previously and related variations

The EMBASE and PUBMED databases were searched for literature review. The clinical phenotypes and associated genotypes of these MTS cases were summarized. Then, comparisons of the types of genotype and onset times of different clinical characteristics were analysed.

## Results

### Clinical multiple-discipline evaluations

The proband of Family 1 (Fig. 1a) was found to have hearing impairment from age three. Otological examinations, including DPOAE, ABR, CM, PTA and Phmax, were performed on this 7-year-old boy, and the typical auditory neuropathy was illustrated. His parents had normal hearing, while the audiogram of the proband showed severe hearing impairment with profoundly damaged SRS (16 and 12% for the left and right ears, respectively). PTA audiogram on the left side was low-frequency ascending and on the right was mid-frequency U-shaped (Fig. 1b). As shown in Fig. 1c, DPOAE for the proband was normal, and no waves could be detected in ABR testing bilaterally, while CM waves from both ears existed (Fig. 1d). ABR testing on patient II:4 (mother of the proband) showed no abnormal latency or amplitude of ABR waves I, III and V (Additional file 1: Figure S1), as well as normal DPOAE, indicating the presence of outer hair cells in the cochlea (Additional file 2: Figure S2). In addition, waves of oVEMP and cVEMP testing showed normal amplitude and latencies, but decreased function of the horizontal semicircular canal was indicated by the bithermal caloric test (details recorded in Additional file 3: Figure S3 & Additional file 4: Table S1). MRI of the proband (Additional file 5: Figure S4) demonstrated an abnormally small cochlear nerve symmetrically. Regarding dystonia, no abnormal manifestation was observed by neurological examination, nor was any found on electromyography. Regarding optic atrophy, both the proband and his mother had normal visual acuity. Flash and pattern visual-evoked potential testing showed a normal latency and amplitude of P100 in both eyes, and detailed stereoscopic fundoscopy showed normal results (Additional file 6: Figure S5). Perimetry was not available for this young child. The γ-globulin level of the proband was normal.

The probands from Family 2 (Fig. 2a) and Family 3 (Fig. 3a), who were diagnosed with auditory neuropathy (Fig. 2b, and c and Fig. 3b, c, d, e, and f), had similar audiological phenotypes as the proband from Family 1, as well as similar MRI findings and visual acuity. The major difference in the proband from Family 3 was the abnormal γ-globulin level, showing agammaglobulinaemia when tested. The serum immunoglobulins of the proband from Family 2 were 0.30, 1.22 and 0.13 g/l of IgA, IgG and IgM, respectively.

### *TIMM8A* variation detection and analysis

**Fig. 4:**
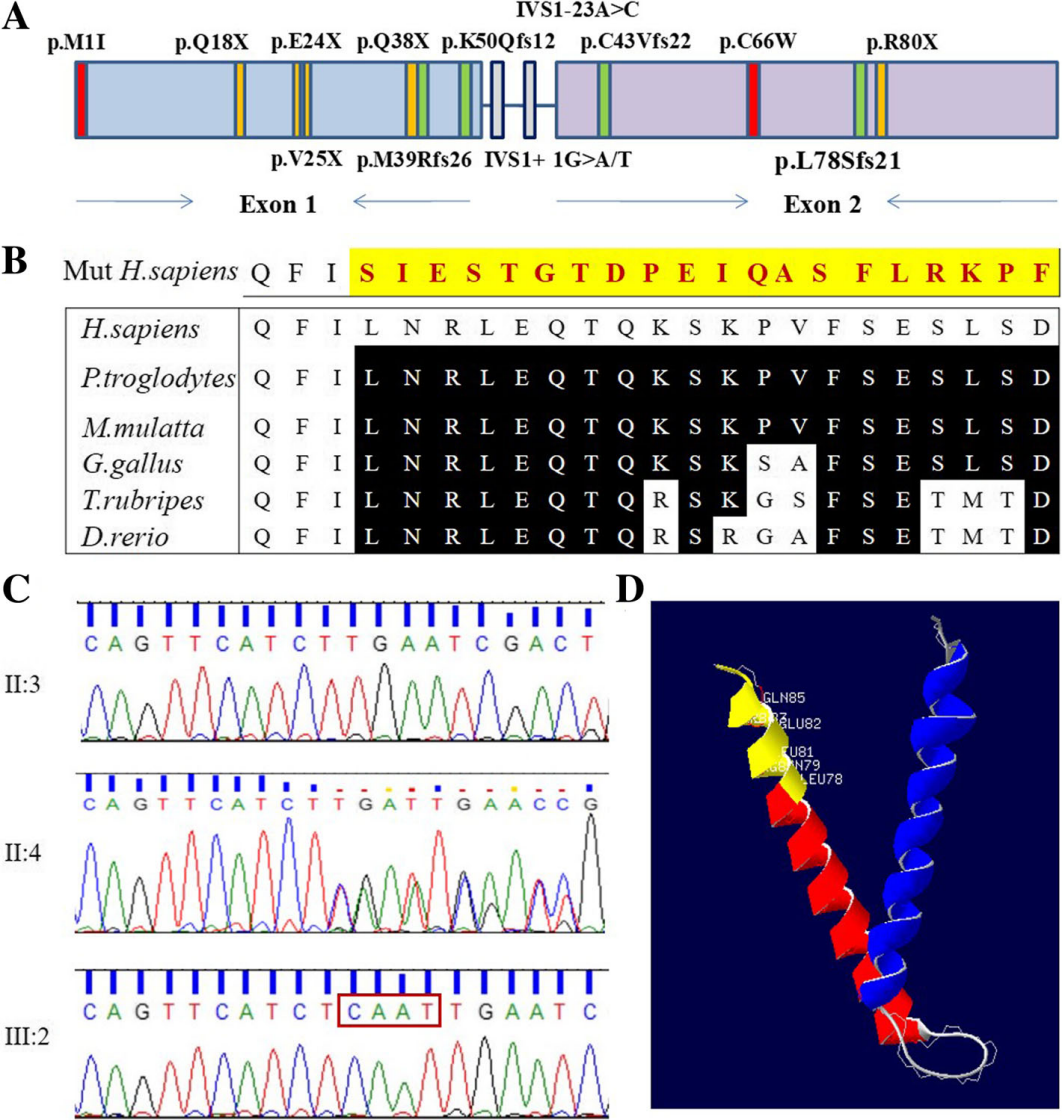
Gene identification in *TIMM8A* of Family 1. **a** General structure of TIMM8A protein showing the positions of variations identified in MTS patients. Red colour presents missense variation, yellow indicates nonsense variation, and green indicates frameshift. **b** Wild-type and mutated amino acid sequences indicating the amino acid changes after variation p.L78Sfs21, and protein alignment showing conservation from amino acids position 78 to 97 across five species. **c** Sequencing chromatograms of TIMM8a showing the insertion in affected individuals (lower panel) compared with that of normal controls (upper panel). The inserted nucleotides are marked by red frames. **d** Three-dimensional structure of TIMM8A wild-type created by SWISS-MODEL. Mutated amino acids are coloured in yellow

We identified a hemizygous variation, c.232_233insCAAT (p.Leu78SerfsX21), in the *TIMM8A* gene (NM_004085) in Family 1 (Fig. 4a and Table 1). This novel variation in the second exon of *TIMM8A* caused one amino acid substitution, leucine to serine at position 78, and a frame shift after that position. That position is highly conserved across species (Fig. 4b). Co-segregation of this variation with the disease in the family was confirmed by using Sanger sequencing, as shown in the Fig. 4c. The c.232_233insCAAT variant was also detected in the proband’s mother with normal hearing, but it was absent in his father. This variation was not found in the 1000 Genomes Project, ExAC 65,000 exome allele frequency data or 1751 ethnicity-matched controls, which further supported its pathogenicity. By using SWISS-MODEL, a three-dimensional molecular structure was built to locate the mutated scope on the second exon of *TIMM8A* (Fig. 4d).

**Table 1:** Overview of known MTS cases on phenotype and related genotype

Year	Source	Affected members, No. of Generations	Phenotypes				Genotypes		
			Onset of Deafness	Onset of Dystonia	Onset of Impaired vision	Psychiatric disorders	TIMM8A Variations		Type
1976	America [20]	3,2	2–6	7	none		NR	NR	NR
1960,1995,1996	Norway [1]	16,5	2–7	7–50	mid-thirties	Peripheral neuropathy, mental deterioration, et al	c.151delT	p.M39Rfs26	Frameshift
1996	America [3]	5,2	NR	NR	none	Mental impairment	c.183del10	p.K50Qfs12	Frameshift
1998	Australia [21]	3,2	2	6–12	12–16	Possible	c.52C > T	p.Q18X	NR
2000	Dutch [22]	1,1	2.5	10	none	Hyper-reflexia, dyspraxia	c.233C > G	p.C66W	missense
2001	Demark [23]	3,5	Early infancy-2	21	14–18	Mildly demented	c.105G > T	p.E24X	nonsense
2001	America [16]	8,4	congenital	Teens-adult	early fifties	Dementia	c.108delG	p.V25X	Frameshift
2001	Japan [24]	5,4	0.5–9	16–30	none	Mild mental impairment	c.238C > T	p.R80X	Nonsense
2003	Germany [25]	1,1	3	28	37	None	c.38G > C	p.M1I	Missense
2004	Italy [26]	1,1	2	19	15	Cognitive decline	complete deletion		
2005	Spain [27]	2,2	4–11	11–20	24	Cognitive decline	IVS1-23A > C		Splice
2006	Spain [28]	2,3	3–7	8	none	Attention deficit and hyperactivity disorder	c.127delT	p.C43Vfs22	Frameshift
2007	UK [29]	1,1	< 1	25	30	None	IVS1+ 1G > A		Splice
2007	Spain [30]	1,1	3	30	15	Distractibility, irritability, and childish manners	c.112C > T	p.Q38X	Nonsense
2007	Czech Republic [17]	2,1	2,5	33	NR	Aggressive behaviour	complete deletion		
	Czech Republic	1,1	4	5	NR	NR	complete deletion		
	Estonia	2,1	2.5,4	NR	NR	Progressing psychomotor retardation	complete deletion		
	African-America	1,1	3	3	NR	NR	complete deletion		
2008	Spain [31]	1,1	4	23	14	Mild mental retardation	IVS1+ 1G > T		Splice
2008	America [19]	1,1	2	NR	NR	NR	exon 1 deletion		
2011	Japan [32]	1,1	1	NR	NR	NR	complete deletion		
	Japan	1,1	1.5	NR	NR	NR	complete deletion		
2013	France [18]	1,1	2.5	8	None	Congenital mental retardation	IVS1+ 1G > A		Splice
2016	Poland [33]	1,1	3	12	None	NR	NR		NR
2016	French-Canadian [34]	4,3	2	NR	NR	progressive psychomotor deterioration	complete deletion		
2018	China	1,2	3	Not yet	Not yet	Not yet	c.232_233insCAAT	p.L78Sfs21	Frameshift
2018	China	1,2	13	Not yet	Not yet	Not yet	c.133_135delGAG	p.Glu45del	Indel
2018	China	1,2	13	Not yet	Not yet	Not yet	arr[hg19] Xq22.1(100,593,213-100,609,547) × 0	/	CNV

**Fig. 5:**
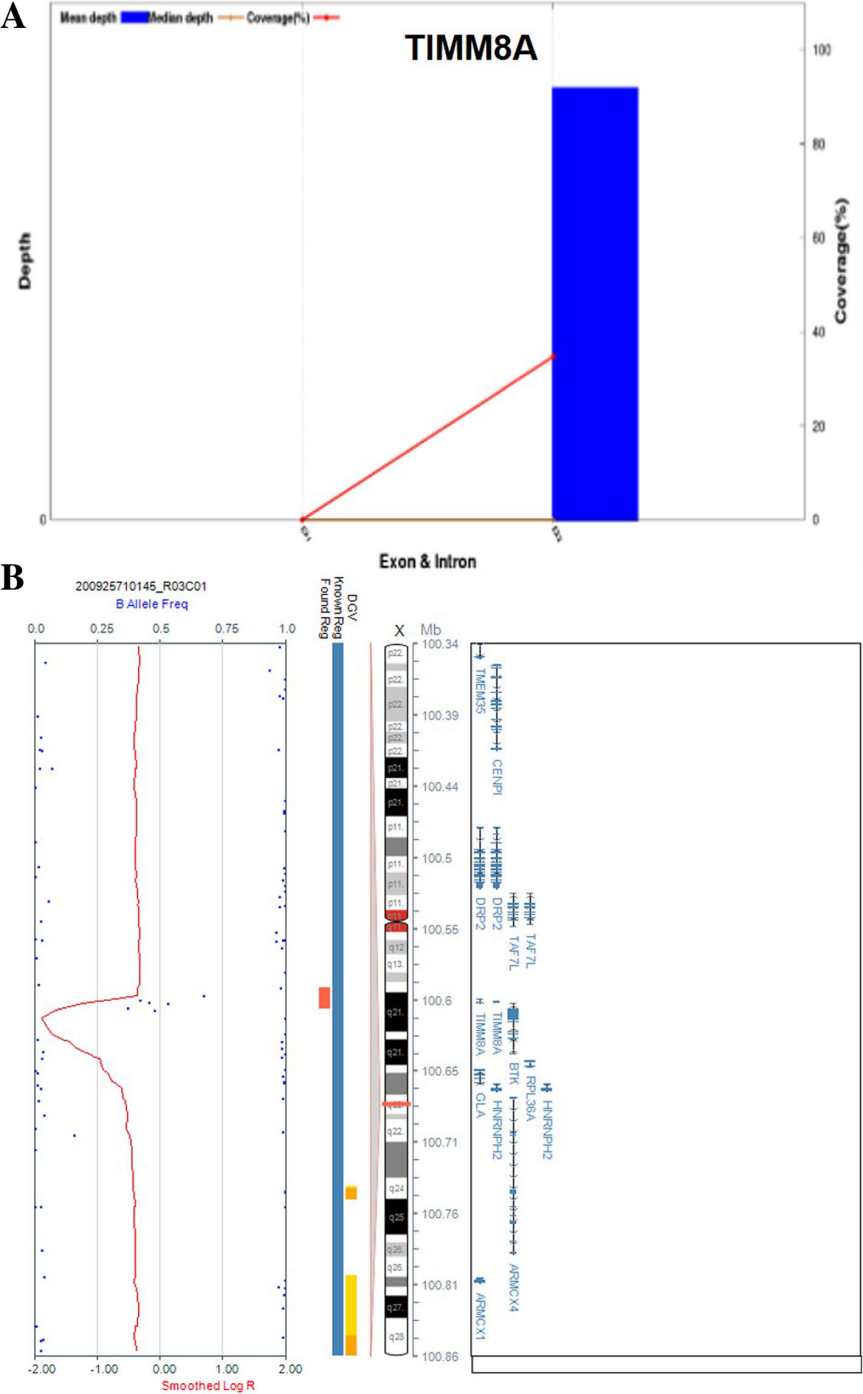
Copy number variation including *TIMM8A* of Family 3. **a** NGS result showing the deletion in the *TIMM8A* gene. **b** Molecular cell karyotype of Xq22.1(100,593,213-100,609,547) × 0 of the proband from Family 3

To further determine the frequency of *TIMM8A* variations in the auditory neuropathy population, we analysed the targeted gene capture and NGS from 167 cases initially diagnosed with auditory neuropathy, with or without other clinical symptoms. We found another two variations: one deletion c.133_135delGAG was identified in Family 2 (Fig. 2d), and a two-exon deletion in the *TIMM8A* gene without a precise location was identified in Family 3 (Fig. 5a). To further confirm the deletion region, a SNP array was performed on the members from Family 3, and the molecular cell karyotype of Xq22.1(100,593,213-100,609,547) × 0 was shown (Fig. 5b), including the TIMM8A and BTK genes. Thus, the proportion of *TIMM8A* variation in the auditory neuropathy population was 1.8% (3/168).

### Overview of known MTS cases on genotype and phenotype

Genotype and phenotype correlation analysis for MTS cases is summarized in Table 1. Worldwide, the 14 previously reported *TIMM8A* abnormalities associated with MTS included missense/nonsense variations, deletions and splicing variants [7]. Therefore, the new insertion variation in this study presented a new genotype for MTS. Regarding phenotype, we observed that three variations in exon 2 did not result in visual loss, whereas other clinical manifestations showed poor correlations with genotypes.

## Discussion

In the present study, we identified three novel *TIMM8A* variations in three Chinese families by applying targeted gene capture to 127 deafness-related genes combined with NGS, as well as a SNP array. The variations of *TIMM8A* were co-segregated in the families and absent in the 1000 Genomes Project, ExAC data and 1751 ethnicity-matched normal controls. According to previous studies [11], the Tim10/DDP family zinc finger domain includes amino acids 22 to 85 of TIMM8A. Both the frameshift variation c.232_233insCAAT and the deletion variation c.133_135delGAG were located in this special domain and resulted in amino acid sequence alterations starting at positions 78 and 45, respectively, impairing the formation of TIMM8A protein. *TIMM8A* belongs to a family of evolutionary conserved proteins assembled into a hetero-hexameric complex with human Tim13 [12]. Thus, these variations in the *TIMM8A* gene might interfere with formation of the heterodimer complex between Tim8 and Tim13.

Auditory neuropathy is a unique hearing dysfunction characterized by absent or abnormal ABR and the presence of OAE and/or CM [13]. Disruption of auditory nerve activity may involve the auditory nerve (postsynaptic auditory neuropathy), inner hair cells and/or the synapses with auditory nerve terminals (presynaptic auditory neuropathy) [14]. The auditory neuropathy in our study belonged to the postsynaptic kind since previous studies on temporal bone histopathology from MTS patients showed a 90–95% loss of cochlear function [15]. Meanwhile, MRI examination of the probands further confirmed the abnormally small cochlear nerve compared with nearby constructions. Somewhat differently, electrophysiologic evaluations of the probands demonstrated the absence of vestibular and optical deficits and dystonia, which may be ascribed to the early stage in the course of MTS. Since few studies have indicated that the carrier female also developed deafness or dystonia [3, 16], the mothers of probands in our study also underwent overall examinations and were assessed as unaffected. Considering MTS is a progressive degeneration [17], long-term follow-up would benefit the patients. Recently, internal globus pallidus (GPi) deep brain stimulation (DBS) was utilized to treat successfully at severe dystonia [18], while cochlear implantation produced little effect [19].

Clinical phenotypes and related known genotypes are overviewed in Table 1, which highlights the high clinical heterogeneity of MTS. All 69 patients reported have hearing impairments within the first decade, but the onset time of dystonia varies, and most occur in the second decade of life. Remarkably, all three probands in this study appeared to be without visual loss at the tested age. The possible explanation for the incomplete optical penetrance in MTS could be related to the damage extent of the structure or function of *TIMM8A*. To our knowledge, this study is the first report of *TIMM8A*-related hearing loss in China, and it is not rare in the auditory neuropathy population, making the case for better understanding the underlying mechanism. Only one patient with CNV in this study showed agammaglobulinaemia. The 16,334-bp deletion including *TIMM8A* and *BTK* identified in Family 3 may be related to contiguous deletion syndrome with the co-existence of X-linked agammaglobulinaemia (XLA) and MTS.

Our original aim in studying these three families was to ask for genetic consultation, as the probands were solely characterized by early onset auditory neuropathy without any signs of dystonia or visual impairment. Genetic diagnosis of MTS for the young patient was confirmed ahead of clinical diagnosis. In fact, it was NGS technology that brought them a clear answer and perspective on the disease. Therefore, targeted capture of deafness genes combined with NGS has become a powerful tool to provide with precise molecular diagnoses and optimal rehabilitation for syndromic hearing-impaired patients as well as accurate genetic counselling for their whole family.

## Conclusion

In conclusion, three novel hemizygous variations of *TIMM8A* were the pathogenic variations in three Chinese families with predicted MTS. Our data extend the variation spectrum of the *TIMM8A* gene and give deeper insight into phenotypes of MTS. Target deafness gene capture and high-throughput NGS increased the diagnosis yield of syndromic genetic hearing loss such as MTS before all symptoms and signs were fully developed.

## Additional files

Additional file 1: **Figure S1.** ABR and CM waves of the proband’s mother (II:4) in Family 1. Normal latency and amplitude of ABR waves I, III and V are shown. (TIF 1369 kb)

Additional file 2: **Figure S2.** DPOAE results of the proband’s mother in Family 1. (TIF 2415 kb)

Additional file 3: **Figure S3.** VEMP waves of the proband in Family 1 showing normal latency and amplitude. (TIF 4272 kb)

Additional file 4: **Table S1.** Vestibular Function Evaluation and results of proband of Family 1. (DOCX 17 kb)

Additional file 5: **Figure S4.** Brain MRI examination of the proband from Family 1. A. Axial view of the cerebellopontine angle and the internal auditory canal (IAC) showing normal anatomy. Two white lines represent the plane prescribed for oblique-plane sagittal images obtained perpendicular to the IAC nerves. B. 3D-fast-spin echo sequence image on oblique plane sagittal from normal age-matched control. Left side demonstrates a normal cochlear nerve (Cn, red arrow), normal-sized IAC, facial (Fn), superior (Vsn) and inferior vestibular nerves (Vin) (yellow arrows). C&D image from proband: abnormally small cochlear nerve (red arrows) in both sides. (TIF 4158 kb)

Additional file 6: **Figure S5.** Visual-evoked potential testing and stereoscopic fundoscopy of the proband from Family 1. (TIF 3098 kb)

## Abbreviations

ABR: Auditory brainstem response
CM: Cochlear microphonicse
CNV: Copy number variation
cVEMP: Cervical vestibular-evoked myogenic potentials
DPOAE: Distortion product otoacoustic emission
ECochG: Electrocochleography
MRI: Magnetic resonance imaging
MTS: Mohr-Tranebjaerg syndrome
NGS: Next generation sequencing
oVEMP: Ocular vestibular-evoked myogenic potentials
PTA: Pure-tone audiometric examination
SRS: Speech recognition score
XLA: X-linked agammaglobulinaemia

## References

[1] Mohr J, Mageroy K (1960). Sex-linked deafness of a possibly new type. Acta Genet Stat Med.

[2] Tranebjaerg L, Schwartz C, Eriksen H, Andreasson S, Ponjavic V, Dahl A (1995). A new X linked recessive deafness syndrome with blindness, dystonia, fractures, and mental deficiency is linked to Xq22. J Med Genet.

[3] Jin H, May M, Tranebjaerg L, Kendall E, Fontan G, Jackson J (1996). A novel X-linked gene, DDP, shows mutations in families with deafness (DFN-1), dystonia, mental deficiency and blindness. Nat Genet.

[4] Tranebjaerg L. Deafness-dystonia-optic Neuronopathy syndrome. In: Pagon RA, Adam MP, Ardinger HH, Wallace SE, Amemiya A, LJH B, Bird TD, Fong CT, Mefford HC, RJH S, et al., editors. GeneReviews(R). Seattle; 1993. 20301395

[5] Arnoult D, Rismanchi N, Grodet A, Roberts RG, Seeburg DP, Estaquier J (2005). Bax/Bak-dependent release of DDP/TIMM8a promotes Drp1-mediated mitochondrial fission and mitoptosis during programmed cell death. Curr Biol.

[6] Roesch K, Hynds PJ, Varga R, Tranebjaerg L, Koehler CM (2004). The calcium-binding aspartate/glutamate carriers, citrin and aralar1, are new substrates for the DDP1/TIMM8a-TIMM13 complex. Hum Mol Genet.

[7] Ha AD, Parratt KL, Rendtorff ND, Lodahl M, Ng K, Rowe DB (2012). The phenotypic spectrum of dystonia in Mohr-Tranebjaerg syndrome. Mov Disord.

[8] Lin YH, Wu CC, Hsu TY, Chiu WY, Hsu CJ, Chen PL (2015). Identification of a novel GATA3 mutation in a deaf Taiwanese family by massively parallel sequencing. Mutat Res.

[9] Gao X, Wang GJ, Yuan YY, Xin F, Han MY, Lu JQ (2014). Novel compound heterozygous mutations in MYO7A associated with usher syndrome 1 in a Chinese family. PLoS One.

[10] Wang H, Wu K, Guan J, Yang J, Xie L, Xiong F, et al. Identification of four TMC1 variations in different Chinese families with hereditary hearing loss. Mol Genet Genomic Med. 2018. 10.1002/mgg3.394. [Epub ahead of print]. 10.1002/mgg3.394 PMC6081220 29654653

[11] Sirrenberg C, Endres M, Folsch H, Stuart RA, Neupert W, Brunner M (1998). Carrier protein import into mitochondria mediated by the intermembrane proteins Tim10/Mrs11 and Tim12/Mrs5. Nature.

[12] Luo LF, Hou CC, Yang WX (2013). Nuclear factors: roles related to mitochondrial deafness. Gene.

[13] Starr A, Picton TW, Sininger Y, Hood LJ, Berlin CI (1996). Auditory neuropathy. Brain.

[14] Wang L, Guan J, Wang H, Lan L, Zhang Q, Zong L (2016). Understanding auditory neuropathy spectrum disorder: a systematic review in transgenic mouse models. Sci China Life Sci.

[15] Bahmad F, Merchant SN, Nadol JB, Tranebjaerg L (2007). Otopathology in Mohr-Tranebjaerg syndrome. Laryngoscope.

[16] Swerdlow RH, Wooten GF (2001). A novel deafness/dystonia peptide gene mutation that causes dystonia in female carriers of Mohr-Tranebjaerg syndrome. Ann Neurol.

[17] Sediva A, Smith CI, Asplund AC, Hadac J, Janda A, Zeman J (2007). Contiguous X-chromosome deletion syndrome encompassing the BTK, TIMM8A, TAF7L, and DRP2 genes. J Clin Immunol.

[18] Cif L, Gonzalez V, Garcia-Ptacek S, James S, Boetto J, Seychelles A (2013). Progressive dystonia in Mohr-Tranebjaerg syndrome with cochlear implant and deep brain stimulation. Mov Disord.

[19] Brookes JT, Kanis AB, Tan LY, Tranebjaerg L, Vore A, Smith RJ (2008). Cochlear implantation in deafness-dystonia-optic neuronopathy (DDON) syndrome. Int J Pediatr Otorhinolaryngol.

[20] Scribanu N, Kennedy C (1976). Familial syndrome with dystonia, neural deafness, and possible intellectual impairment: clinical course and pathological findings. Adv Neurol.

[21] Hayes MW, Ouvrier RA, Evans W, Somerville E, Morris JG (1998). X-linked dystonia-deafness syndrome. Mov Disor.

[22] Tranebjaerg L, Hamel BC, Gabreels FJ, Renier WO, Van Ghelue M (2000). A de novo missense mutation in a critical domain of the X-linked DDP gene causes the typical deafness-dystonia-optic atrophy syndrome. Eur J Hum Genet.

[23] Tranebjaerg L, Jensen PK, Van Ghelue M, Vnencak-Jones CL, Sund S, Elgjo K (2001). Neuronal cell death in the visual cortex is a prominent feature of the X-linked recessive mitochondrial deafness-dystonia syndrome caused by mutations in the TIMM8a gene. Ophthalmic Genet.

[24] Ujike H, Tanabe Y, Takehisa Y, Hayabara T, Kuroda S (2001). A family with X-linked dystonia-deafness syndrome with a novel mutation of the DDP gene. Arch Neurol.

[25] Binder J, Hofmann S, Kreisel S, Wohrle JC, Bazner H, Krauss JK (2003). Clinical and molecular findings in a patient with a novel mutation in the deafness-dystonia peptide (DDP1) gene. Brain.

[26] Pizzuti A, Fabbrini G, Salehi L, Vacca L, Inghilleri M, Dallapiccola B (2004). Focal dystonia caused by Mohr-Tranebjaerg syndrome with complete deletion of the DDP1 gene. Neurology.

[27] Ezquerra M, Campdelacreu J, Munoz E, Tolosa E, Marti MJ (2005). A novel intronic mutation in the DDP1 gene in a family with X-linked dystonia-deafness syndrome. Arch Neurol.

[28] Aguirre LA, del Castillo I, Macaya A, Meda C, Villamar M, Moreno-Pelayo MA (2006). A novel mutation in the gene encoding TIMM8a, a component of the mitochondrial protein translocase complexes, in a Spanish familial case of deafness-dystonia (Mohr-Tranebjaerg) syndrome. Am J Med Genet A.

[29] Kim HT, Edwards MJ, Tyson J, Quinn NP, Bitner-Glindzicz M, Bhatia KP (2007). Blepharospasm and limb dystonia caused by Mohr-Tranebjaerg syndrome with a novel splice-site mutation in the deafness/dystonia peptide gene. Mov Disord.

[30] Blesa JR, Solano A, Briones P, Prieto-Ruiz JA, Hernandez-Yago J, Coria F (2007). Molecular genetics of a patient with Mohr-Tranebjaerg syndrome due to a new mutation in the DDP1 gene. NeuroMolecular Med.

[31] Aguirre LA, Perez-Bas M, Villamar M, Lopez-Ariztegui MA, Moreno-Pelayo MA, Moreno F (2008). A Spanish sporadic case of deafness-dystonia (Mohr-Tranebjaerg) syndrome with a novel mutation in the gene encoding TIMM8a, a component of the mitochondrial protein translocase complexes. Neuromuscul Disord.

[32] Arai T, Zhao M, Kanegane H, van Zelm MC, Futatani T, Yamada M (2011). Genetic analysis of contiguous X-chromosome deletion syndrome encompassing the BTK and TIMM8A genes. J Hum Genet.

[33] Dulski J, Schinwelski M, Mandat T, Pienczk-Reclawowicz K, Slawek J (2016). Long-term follow-up with video of a patient with deafness-dystonia syndrome treated with DBS-GPi. Stereotact Funct Neurosurg.

[34] Shaker M, Lorigiano TH, Vadlamudi A (2016). Xq22.1 contiguous gene deletion syndrome of X-linked agammaglobulinemia and Mohr-Tranebjaerg syndrome. Ann Allergy Asthma Immunol.

