# Supplementary figures and images for: Phenotype prediction of Mohr-Tranebjaerg syndrome (MTS) by genetic analysis and initial auditory neuropathy
Source: BMC Med Genet. 2019 Jan 11;20:11. doi: 10.1186/s12881-018-0741-3 (PMC6330410; doi:10.1186/s12881-018-0741-3)

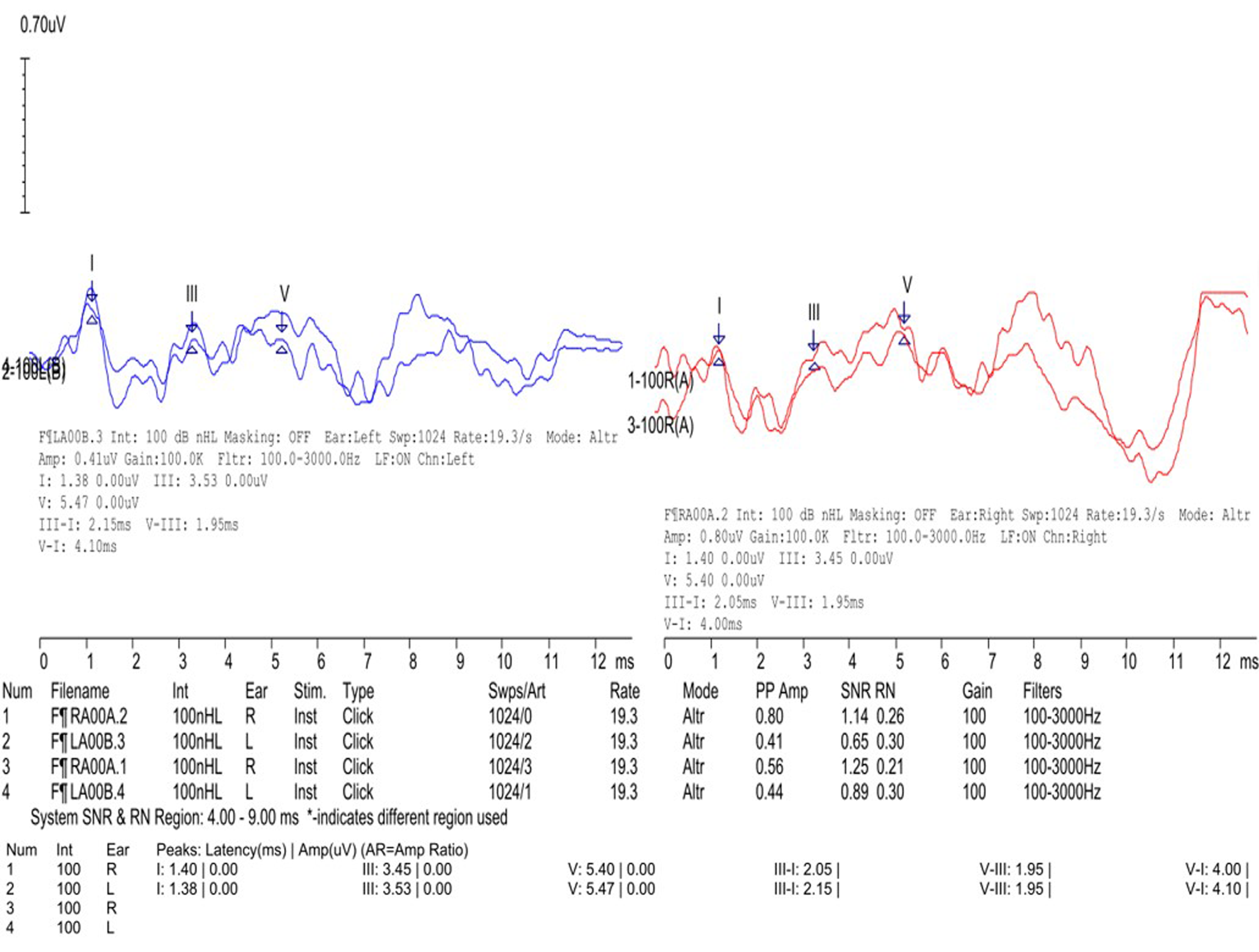

Supplement: Supplementary file 1 — Figure S1. ABR and CM waves of the proband’s mother (II:4) in Family 1. Normal latency and amplitude of ABR waves I, III and V are shown. (TIF 1369 kb) [file 12881_2018_741_MOESM1_ESM.tif]

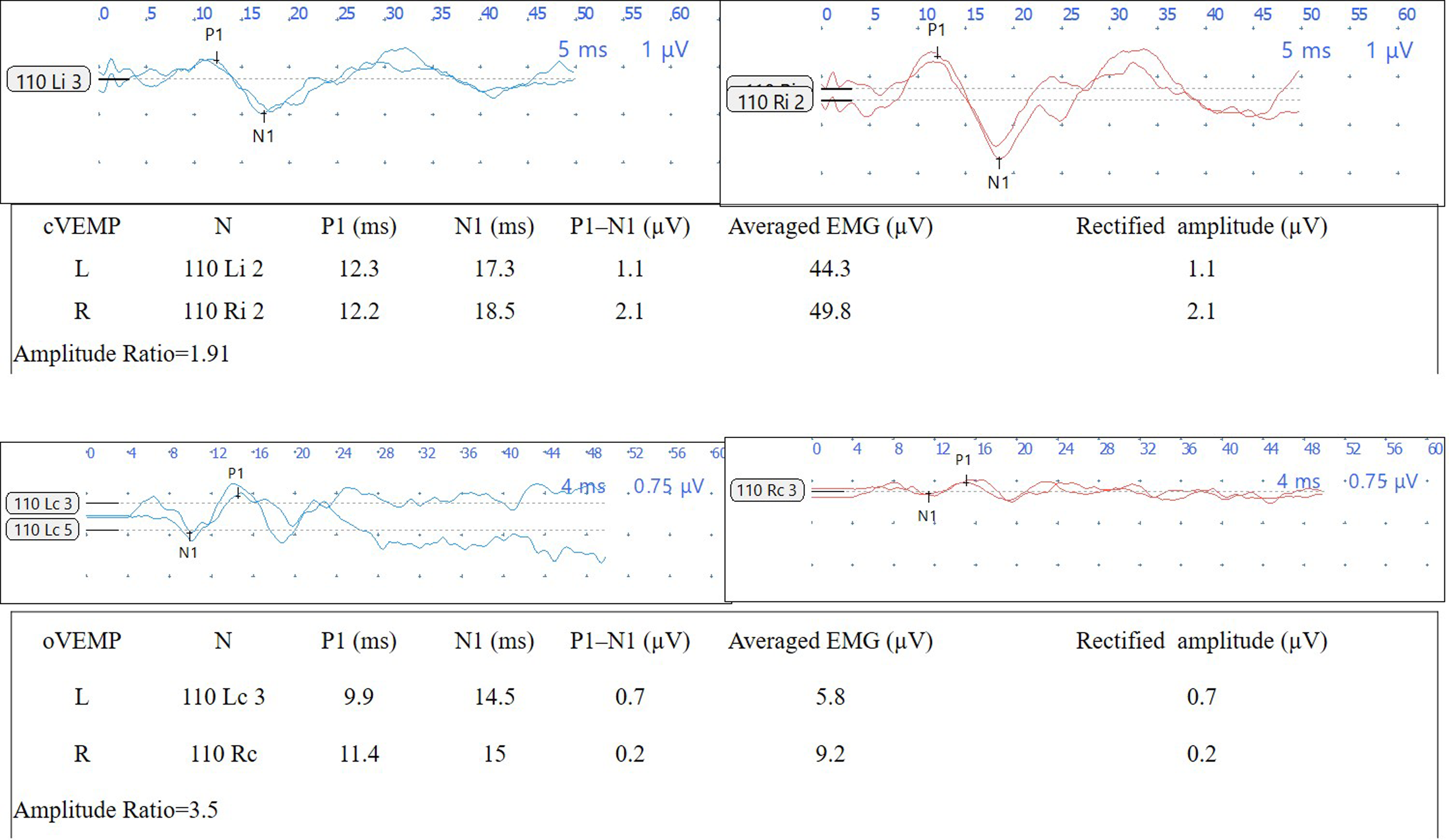

Supplement: Supplementary file 2 — Figure S2. DPOAE results of the proband’s mother in Family 1. (TIF 2415 kb) [file 12881_2018_741_MOESM2_ESM.tif]

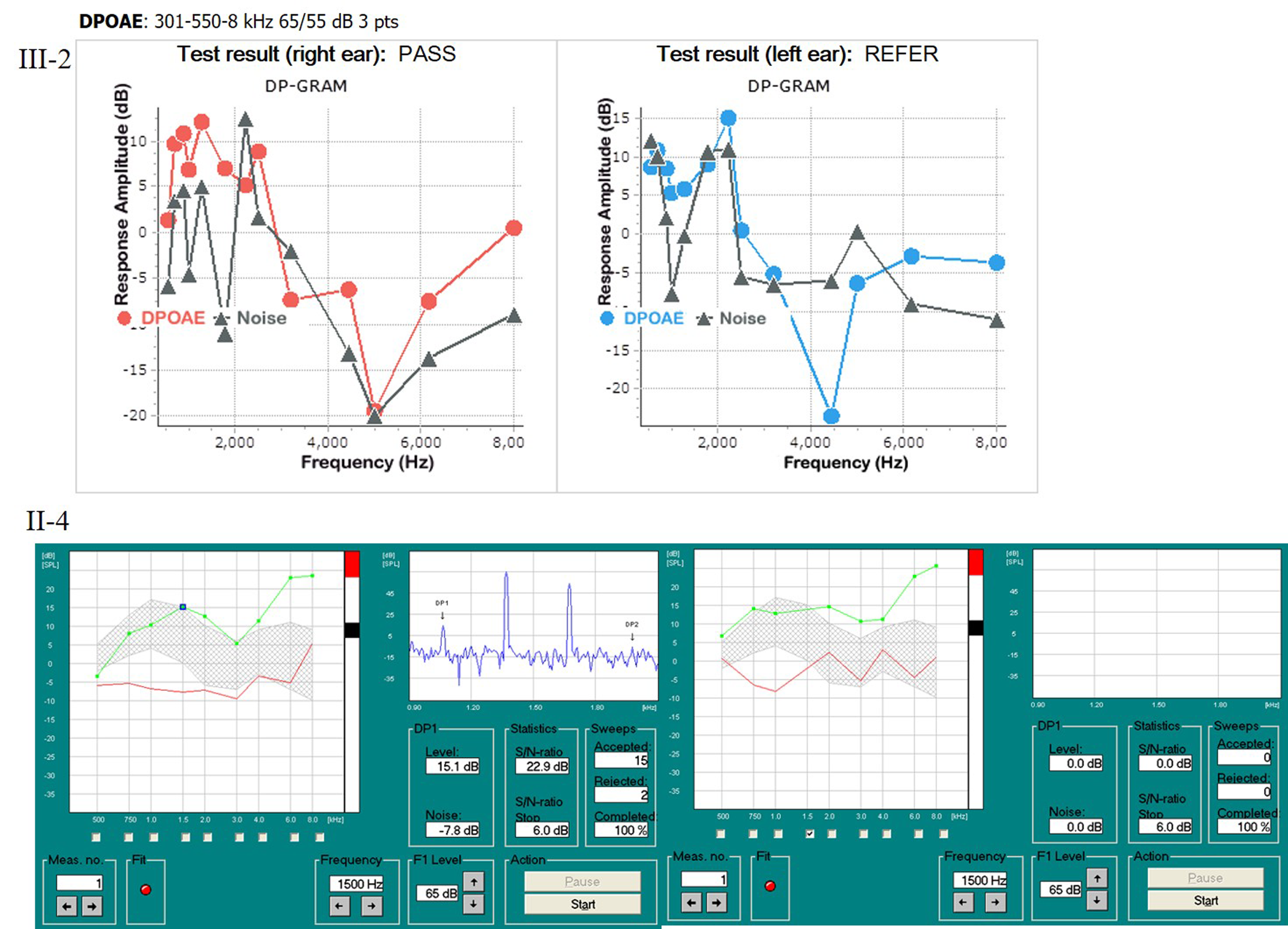

Supplement: Supplementary file 3 — Figure S3. VEMP waves of the proband in Family 1 showing normal latency and amplitude. (TIF 4272 kb) [file 12881_2018_741_MOESM3_ESM.tif]

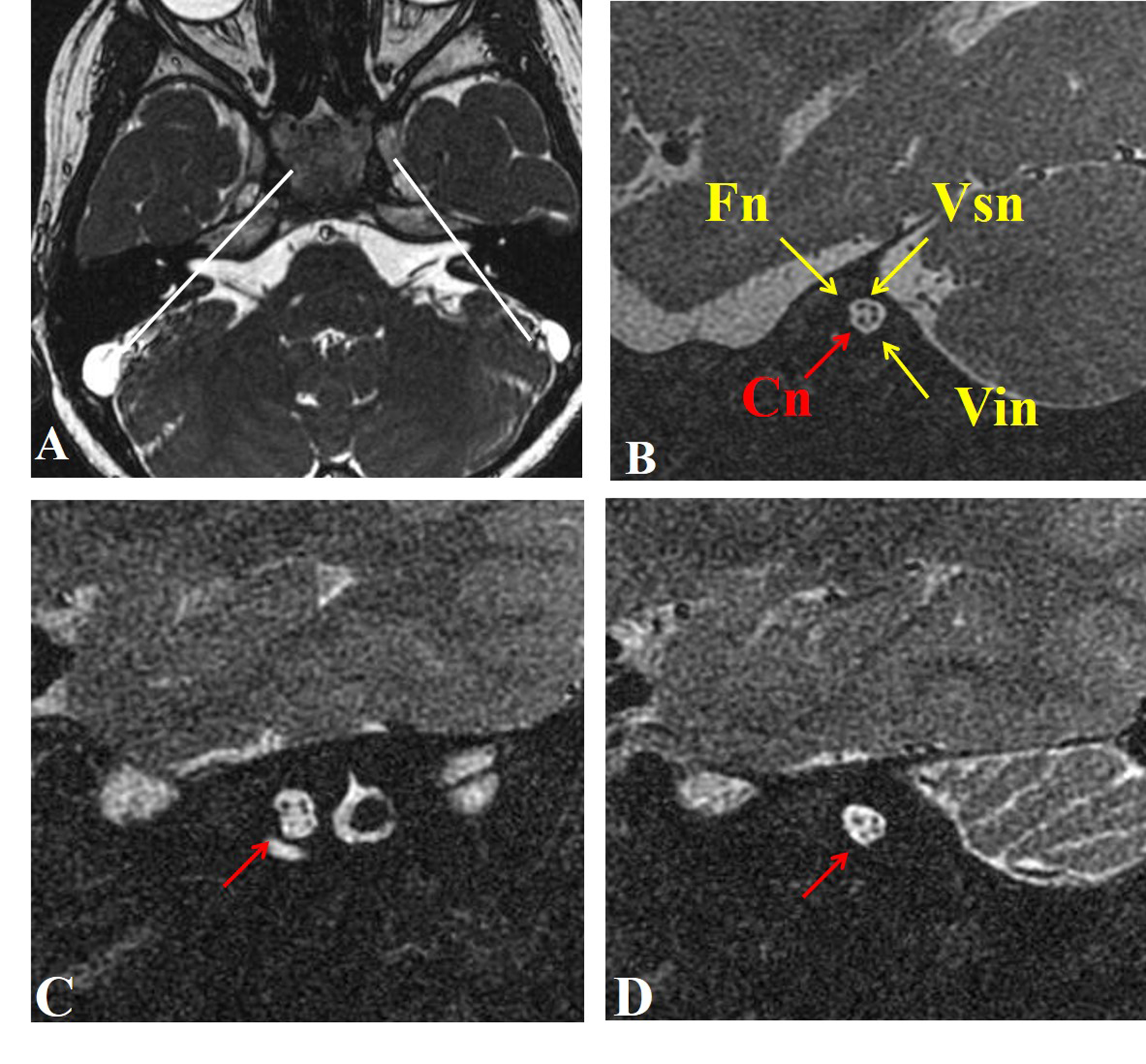

Supplement: Supplementary file 5 — Figure S4. Brain MRI examination of the proband from Family 1. A. Axial view of the cerebellopontine angle and the internal auditory canal (IAC) showing normal anatomy. Two white lines represent the plane prescribed for oblique-plane sagittal images obtained perpendicular to the IAC nerves. B. 3D-fast-spin echo sequence image on oblique plane sagittal from normal age-matched control. Left side demonstrates a normal cochlear nerve (Cn, red arrow), normal-sized IAC, facial (Fn), superior (Vsn) and inferior vestibular nerves (Vin) (yellow arrows). C&D image from proband: abnormally small cochlear nerve (red arrows) in both sides. (TIF 4158 kb) [file 12881_2018_741_MOESM5_ESM.tif]

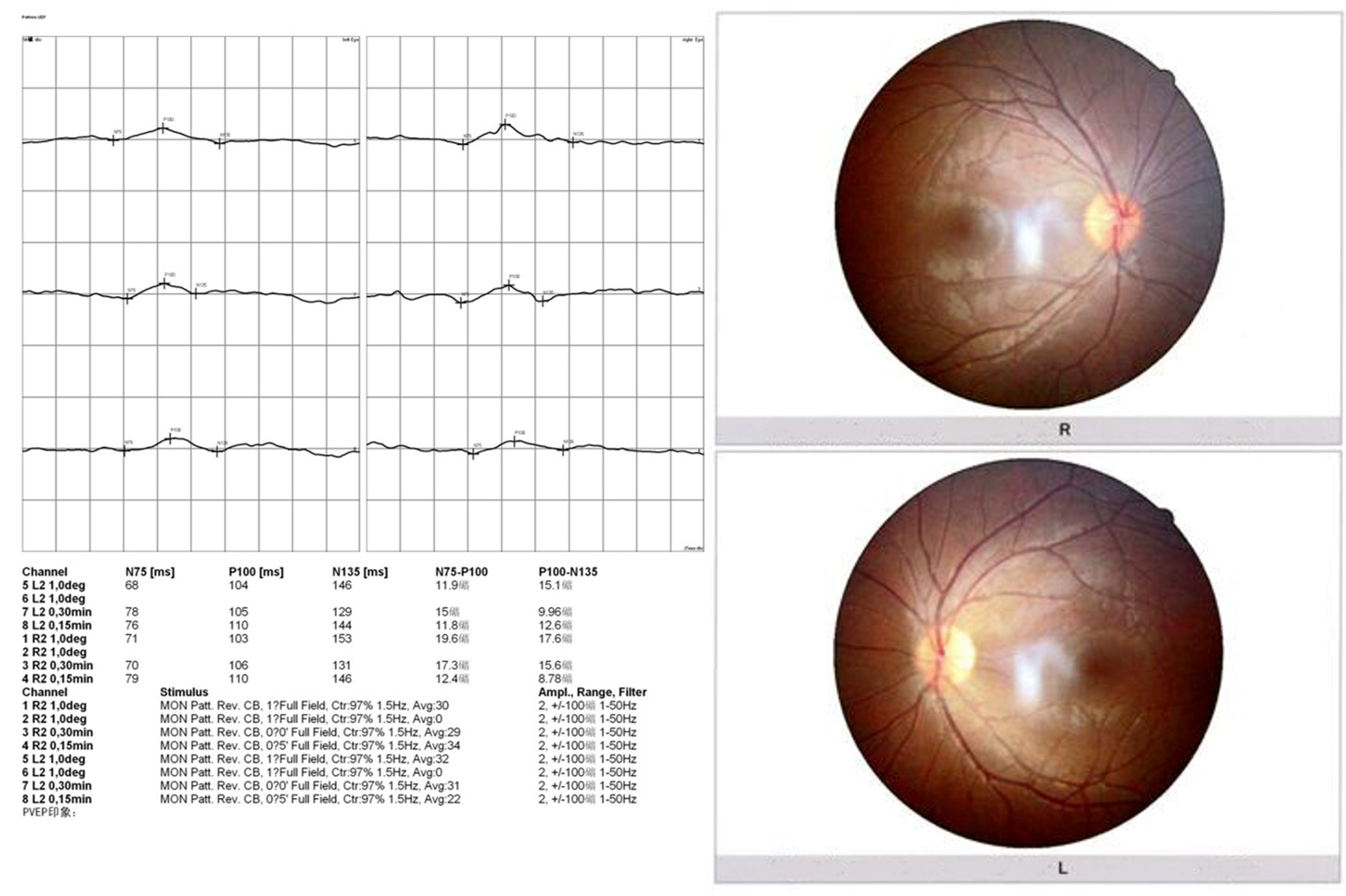

Supplement: Supplementary file 6 — Figure S5. Visual-evoked potential testing and stereoscopic fundoscopy of the proband from Family 1. (TIF 3098 kb) [file 12881_2018_741_MOESM6_ESM.tif]
