# Supplementary material for: Phenotype prediction of Mohr-Tranebjaerg syndrome (MTS) by genetic analysis and initial auditory neuropathy
Source: BMC Med Genet. 2019 Jan 11;20:11. doi: 10.1186/s12881-018-0741-3 (PMC6330410; doi:10.1186/s12881-018-0741-3)
Supplement: Supplementary file 4 — Table S1. Vestibular Function Evaluation and results of proband of Family 1. (DOCX 17 kb) [file 12881_2018_741_MOESM4_ESM.docx]

Additional file 4: Table S1. Vestibular Function Evaluation and results of proband of Family 1

| Oculomotor function tests | ocular dysmetria test | | | | normal |
| --- | --- | --- | --- | --- | --- |
|  | smooth pursuit test | | | | type Ⅱ-Ⅲ |
|  | optokinetic nystagmus | | | | normal |
|  | gaze test | | | | negative |
| Positional nystagmus | spontaneous nystagmus | | | | none |
|  | head-shaking nystagmus | | | | none |
|  | positional nystagmus (supine, lateral position, hanging head, lateral hanging head) | | | | none |
| Positioning nystagmus | Dix-Hallpike | | | | negative |
|  | Roll | | | | negative |
| Bithermal caloric test |  |  | direction | slow-phase velocity | fixation suppression |
|  | 44 | R |  | 0 |  |
|  |  | L | → | 9 | ＋ |
|  | 30 | R |  | 0 |  |
|  |  | L | ← | 13 | ＋ |
